# First Report of Anthelmintic Resistance in Gastrointestinal Nematodes in Goats in Romania

**DOI:** 10.3390/ani11102761

**Published:** 2021-09-22

**Authors:** Adrian Valentin Potârniche, Marcin Mickiewicz, Diana Olah, Constantin Cerbu, Marina Spînu, Attila Hari, Adriana Györke, Agata Moroz, Michał Czopowicz, Marián Várady, Jarosław Kaba

**Affiliations:** 1 Department of Infectious Diseases, Faculty of Veterinary Medicine, University of Agricultural Sciences and Veterinary Medicine of Cluj-Napoca, Mănăștur Str. 3-5, 400372 Cluj, Romania; adrian.potarniche@usamvcluj.ro (A.V.P.); diana.olah@usamvcluj.ro (D.O.); constantin.cerbu@usamvcluj.ro (C.C.); marina.spinu@usamvcluj.ro (M.S.); 2 Division of Veterinary Epidemiology and Economics, Institute of Veterinary Medicine, Warsaw University of Life Sciences, Nowoursynowska Str. 159c, 02-776 Warsaw, Poland; moroz.agata@gmail.com (A.M.); michal_czopowicz@sggw.edu.pl (M.C.); jaroslaw_kaba@sggw.edu.pl (J.K.); 3 Department of Physiology, Faculty of Veterinary Medicine, University of Agricultural Sciences and Veterinary Medicine of Cluj-Napoca, Mănăștur Str. 3-5, 400372 Cluj, Romania; attila.hari@yahoo.com; 4 Department of Parasitology and Parasitic Diseases, Faculty of Veterinary Medicine, University of Agricultural Sciences and Veterinary Medicine of Cluj-Napoca, Mănăștur Str. 3-5, 400372 Cluj, Romania; adriana.gyorke@usamvcluj.ro; 5 Institute of Parasitology Slovak Academy of Sciences, Hlinkova 3, 04001 Košice, Slovakia; varady@saske.sk

**Keywords:** anthelmintic resistance, goats, benzimidazoles, macrocyclic lactones

## Abstract

**Simple Summary:**

Anthelmintic resistance (AR) is a serious threat to animal health and has a major economic impact worldwide due to production and financial losses. Currently, there are three classes of anthelmintics most commonly used in small ruminants: the benzimidazoles (BZs), macrocyclic lactones (MLs) and cholinergic agonists (especially levamisole; LEV). The widespread use of those products has led to the emergence of drug-resistant parasite strains. In the present study, we describe for the first time a case of resistance to anthelmintics in goats in Romania. Resistance was detected and confirmed for two chemical groups of anthelmintics (MLs and BZs) by in vivo faecal egg count reduction test (FECRT) and in vitro methods: the egg hatch test (EHT) and larval development test (LDT). Considering the increasing prevalence of AR in goat herds in Europe and around the globe, we believe that the findings of our study on AR in goats in Romania do not represent a singular event and could hence be just the noticeable part of a much wider issue.

**Abstract:**

Currently, there are three classes of anthelmintics most commonly used in small ruminants: the benzimidazoles (BZs), macrocyclic lactones (MLs) and cholinergic agonists (especially levamisole; LEV). The widespread use of those products has led to the emergence of drug-resistant parasite strains which represents a serious threat to the livestock industry. In the present study, we describe for the first time a case of resistance to anthelmintics in goats in Romania. The study was carried out in 2021 in a dairy goat herd from the Transylvania region. Two types of diagnostic methods were used to confirm anthelmintic resistance (AR). First, the faecal egg count reduction test (FECRT), an in vivo AR diagnostic method, was used to evaluate the efficacy of eprinomectin (EPM). The results of this test were analysed applying two different calculative methods that are used only in treated animals (without the control group). Furthermore, two in vitro methods were used: the egg hatch test (EHT) for the detection of resistance to BZs, and the larval development test (LDT) for detection of resistance to all three classes of anthelmintics. The results of FECRT indicate the resistance of gastrointestinal nematodes (GINs) to EPM in both calculative methods (FECR_1_ = −88% and FECR_2_ = −202%). In addition, the results obtained for ivermectin aglycone (IVM-AG) in LDT also indicate resistance to drugs from MLs group, especially avermectins. Similarly, the results of in vitro methods (EHT and LDT) indicate resistance to BZs in this herd. LEV was the only drug that stopped the development of L3 larvae 100% (LDT). *H. contortus* was the only nematode species found in coproculture after EPM treatment. Furthermore, *H. contotus* L3 larvae was the only species found in the wells with the highest concentrations of thiabendazole (TBZ) and IVM-AG in LDT. This suggests that resistance to both BZs and MLs was present for that species.

## 1. Introduction

Parasitic infections, especially those caused by gastrointestinal nematodes (GINs), are one of the main causes of economic losses in goat production worldwide [1]. Therefore, the infection with GINs, such as *Haemonchus contortus*, *Trichostrongylus* spp. and *Teladorsagia* spp. threatens the profitability and sustainability of goat production [1,2]. Their control is mainly based on the use of anthelmintics [3]. Currently, there are three classes of anthelmintics most commonly used in small ruminants: the benzimidazoles (BZs), macrocyclic lactones (MLs) and cholinergic agonists (especially levamisole; LEV) [4,5]. The widespread use of those products has led to the emergence of drug-resistant parasite strains [1]. Resistance may arise to one anthelmintic class, or even to several or all classes of anthelmintics, referred to as multidrug resistance (MDR). The distribution of anthelmintic resistance seems to correspond to the popularity of anthelmintic classes used in veterinary practice [6,7]. Therefore, the increase in MDR among GINs and the lack of sufficiently effective alternative methods of control and prophylaxis of parasitic infections are an increasing threat to small ruminant health and production around the globe [8,9]. For example, resistance of GINs to one or more anthelmintic classes in goats has so far been described in many European countries including Great Britain [10], the Netherlands [11], Spain [12], Italy [13], Lithuania [14], France [15,16], Switzerland [17], Denmark [18], Norway [19], Germany [20], Slovakia [21], Czech Republic [22], and Poland [7,23,24,25]. The situation is similar in goat herds outside of Europe as described in USA [26], South Africa [27], Kenya [28], Cuba [29], Malaysia [30], India [31], Australia [32], and New Zealand [33].

Currently, the detection methods used for anthelmintic resistance (AR) are grouped into three categories: a) in vivo methods, mainly represented by the faecal egg count reduction test (FECRT); b) in vitro methods, particularly the egg hatch test (EHT) and the larval development test (LDT), and c) molecular biology techniques. The recommended standard AR detection method by the World Association for Advance in Veterinary Parasitology (WAAVP), is faecal egg count reduction test (FECRT) [4,5]. However, this method is time-consuming, variation between animals is high, and the pharmacokinetics of the anthelmintic in the host may negatively influence the results [21]. Therefore, interest was directed towards in vitro tests for detecting AR. These provide comparable and reliable results and can detect low proportions of resistant worms in a population, with the sensitivity that has potential in determining resistance with field tests [34,35,36,37].

In Romania, AR has been so far documented only in horses [38]. In the present study, we aim to describe for the first time a case of AR in goats in Romania.

## 2. Materials and Methods

### 2.1. Animals

The study was carried out in a dairy goat herd from Transylvania region, Romania in 2021. The herd consisted of 3 adult males, 38 adult females, and 20 kids. The goats were housed in a 100 m^2^ wooden barn. The herd was established in 2016 by purchasing a few young adult females (<6 months) of Carpathian breed from the same village where the herd is located. In 2019, the owner bought 20 French Alpine adult goats (males and females) from Alba County, Transylvania, the same County where the herd is located. Then, the herd relied on their own replacement goats. The animals were grazed from May to November for 8–10 h per day on a large pasture (4 ha) fenced with electric wire. Additionally, they were fed on alfalfa hay and during milking; the goats received concentrates (oat and corn). Mineral supplements consisted of mineral blocks with selenium. The medical records of the herd attest that all adult goats had been regularly dewormed (starting from 2017) twice per year as follows: using ivermectin 0.2 mg/kg by subcutaneous injection (Evomec 10 mg/mL, Pasteur, Romania) in the autumn, and using albendazole 10 mg/kg per os (Gardal 10%, Intervet, Romania) in the spring. Despite regular deworming, in the spring of 2021, several adult goats presented weight loss, diarrhoea, and submandibular oedema. No parasitological examinations were previously performed, except a simple flotation exam which revealed infestation with GINs (strongyle type eggs).

### 2.2. Sampling and Laboratory Analysis

#### 2.2.1. Faecal Egg Count Reduction Test (FECRT)

In the spring of 2021, ten adult goats (>6 months) were randomly selected for this study. The selected animals had not been treated with any anthelmintics for at least 8 weeks prior to the study. They were kept indoors during the study. No control group was used in the present study. The FECRT was performed at the Department of Parasitology and Parasitic Diseases, Faculty of Veterinary Medicine, University of Agricultural Sciences and Veterinary Medicine of Cluj-Napoca, according to the guidelines of the World Association for the Advancement of Veterinary Parasitology (W.A.A.V.P.) [4,5]. Before treatment, each goat was weighed on an electronic scale. The goats received the recommended doses of eprinomectin (EPM) 1 mg/kg pour on [39] (Eprinex multi 5 mg/mL, Boehringer Ingelheim, France). The drug was administered by local veterinarians in the presence of the owner. Faecal samples (about 10 g) were taken straight from the rectum on the day of the treatment (day 0) and 14 days after the treatment (day 14). The samples were brought to the lab at 4 °C and examined within 24 h after collection by the modified McMaster procedure with an analytical sensitivity of 50 epg [4,5]. Coproculture was prepared by mixing 5 g of faeces collected from each animal before and after the treatment and pooled in one sample. After incubation, third stage larvae (L3) from each pool were identified at the genus level following the method described by van Wyk and Mayhew [40]. Differentiation between *Trichostrongylus* spp. and *Teladorsagia* spp. was performed by comparing specific morphological features of GIN species using 400x light microscope magnification after exsheathement of the L3 larvae in 3.5% sodium hypochlorite solution.

#### 2.2.2. Egg Hatch Test (EHT)

The EHT was performed 10 weeks after FECRT at the Division of Veterinary Epidemiology and Economics, Institute of Veterinary Medicine, Warsaw University of Life Sciences, Warsaw, Poland. The test was carried out according to Coles et al. [4,5]. Fresh faecal samples from 10 goats were collected by the farmer, pooled, homogenised and then tap water was added to fill at least 100 mL bottles in order to provide anaerobic conditions [41]. The pooled sample was sent to the laboratory and performed within 72 h after collection. Helminth eggs were extracted from the pooled sample by sieving through sieves of 250, 100 and 25 μm, and centrifuged (3000 rpm, 10′) in Sheather’s sugar solution. The suspension of eggs thus obtained was suspended in deionised water. The thiabendazole stock solution (TBZ; Sigma-Aldrick, Merck, Germany) was prepared by dissolving in pure dimethyl sulfoxide (DMSO; Sigma-Aldrick, Merck, Germany) as described by von Samson-Himmelstjerna et al. [42]. Subsequently, five concentrations of TBZ solution (0.05, 0.1, 0.3, 0.5 and 1.0 μg/mL) were prepared and added to the 24-well plate. The final concentration was set by adding 10 μL of TBZ solution into 1.99 mL of the eggs suspension (100 eggs/mL). Each concentration of TBZ was tested in duplicate. DMSO without any anthelmintic served as the control. The 24-well plate was sealed to prevent drying and incubated at 25 °C for 48 h, and then stained with 10 μL of Lugol’s iodine per each well. The wells were subsequently examined under the inverted microscope (Olympus, CKX53, Poland) at 100× magnification and the number of unhatched eggs and first-stage larvae (L1) in each well were counted [5].

#### 2.2.3. Larval Development Test (LDT)

LDT was performed simultaneously to the EHT at the Division of Veterinary Epidemiology and Economics, Institute of Veterinary Medicine, Warsaw University of Life Sciences, Warsaw, Poland. The test was performed according to Hubert and Kerboeuf [43], with further modifications made by Várady et al. [44]. The collection, storage, and extraction of eggs were the same as described above for EHT. Stock solutions of thiabendazole (TBZ; Sigma-Aldrich, Merck, Germany) and ivermectin aglycone (IVM-AG, Tebu-bio, France) were prepared by dissolving in pure DMSO (Sigma-Aldrich, Merck, Germany). Stock solution of levamisole (LEV; Sigma-Aldrich, Merck, Germany) was prepared by dissolving in deionised water. Subsequently, 12 concentrations for each drug were prepared by serially diluting (1:2) in DMSO (TBZ and IVM-AG) and deionised water (LEV). These concentrations ranged from 0.0006 to 1.28 μg/mL for TBZ, from 0.084 to 173.6 ng/mL for IVM-AG, and from 0.02 to 32 μg/mL for LEV. The test was performed in a 96-well cell culture plate (Sarstedt, Germany) with 150 μL culture medium containing (all in one test plate) 10 μL of TBZ, IVM-AG, LEV or DMSO solution and deionised water (control wells), 110 μL of deionised water, 20 μL of nutritive medium (yeast extract with Earle’s Balanced Salt Solution) [41], and another 10 μL of egg suspension (roughly 100 eggs) containing Amphotericin B (Sigma-Aldrich, Merck, Germany) at a concentration of 5 μg/mL. Each concentration of TBZ, IVM-AG and LEV was tested in duplicate. Next, the plate was incubated for 7 days at 25 °C and then stained with 10 μL of Lugol’s iodine per each well to stop larval development. The unhatched eggs and L1-L3 larvae from all wells were examined using an inverted microscope (Olympus, CKX53, Poland). Infectious L3 in the wells were examined for morphological features and identified at the genus/species level as described by van Wyk and Mayhew [40].

### 2.3. Data Analysis

#### 2.3.1. FECRT

Faecal egg count results were presented as the arithmetic mean (± standard deviation, SD), median (interquartile range, IQR), and range. The 95% confidence intervals (CI 95%) for percentages were calculated using the Wilson score method.

The percent reduction in faecal egg count (FECR%) was calculated using two different formulae.

(a) FECR_1_ = 100 × (1 − [T_1_/T_0_]) [45], where T_0_ and T_1_ represent the arithmetic mean of eggs per gram (EPGs) of the treated group before (day 0) and after treatment (day 14).
(b) FECR_2_ = (1/*n*)Σ(100 × (1 − [Ti_1_/Ti_0_]) [46], where Ti_0_ and Ti_1_ represent the EPGs (before and after treatment) in host *i* from a total *n* hosts. In this case, each host serves as its own control.

Anthelmintic resistance was considered in both methods if FECR% was less than 95% [45,46] and only for the first method also when simultaneously the lower limit of 95% confidence interval (CI 95%) was lower than 90% [5,45].

#### 2.3.2. EHT

Results of the EHT were determined as the TBZ concentration required to inhibit 50% or 99% of the eggs from hatching (inhibition concentration, IC_50_) and the number of hatched eggs at a concentration of 0.1 μg/mL of TBZ, since the results of Coles et al. [5] suggest that this discrimination concentration (DC) prevents hatching of 99% of susceptible eggs. Thus, the percentage of hatched eggs is a direct measure of the percentage of BZ-resistant eggs in the sample [37]. The number of hatched eggs was corrected for natural mortality from control wells (corrected percentage inhibition, cPI%) [5]. The concentrations of TBZ were log transformed, and an S-shaped dose–response curve was fitted by converting cPI% to their probits, defined as normal equivalent deviates increased by 5 to avoid calculating with negative numbers [46]. Benzimidazole resistance was considered if the IC_50_ value was above the DC of 0.1 μg/mL and hatching of larvae was observed [5].

#### 2.3.3. LDT

The arithmetic mean percentage of developing larvae in the tested wells (PDT) at each anthelmintic concentration was corrected by the percentage of developing larvae in the control wells (PDC) using the formula below:

cPD%  =  PDT/PDC, where cPD% stood for the corrected percentage of larvae developing in the tested wells.

The four-parameter logistic curve was used to estimate the concentration of each anthelmintic that inhibited development of 50% (median lethal concentration, LC_50_), and 99% (LC_99_) of larvae [47]. The results of LDT were interpreted with respect to the DC of the anthelmintic agent which was defined as the concentration of anthelmintic at which the development of at least 99% of susceptible larvae (adjusted by the PDC) would have been inhibited [5,47]. In this study, the following DC were applied: 0.08 μg/mL for TBZ [21], 21.6 ng/mL [14,48] for IVM-AG, and 2.0 μg/mL for LEV [49]. Statistical analysis was performed in TIBCO Statistica 13.3.0 (TIBCO Software Inc., Palo Alto, CA, USA).

## 3. Results

### 3.1. FECRT

**Figure 1:**
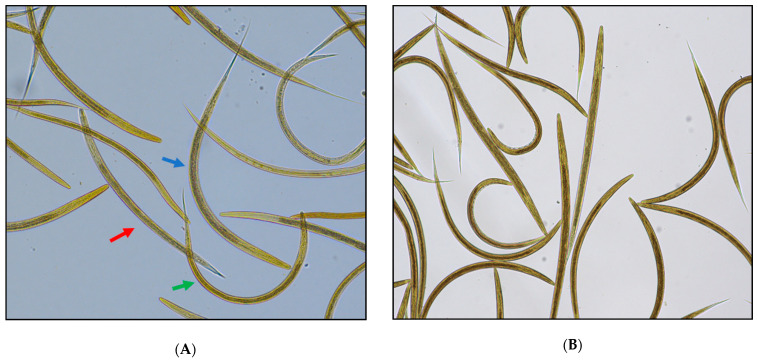
(**A**) Coproculture before treatment: *H. contortus* (green arrow), *T. circumcincta* (red arrow) and *Oesophagostomum* spp. (blue arrow) third-stage larvae (L3) in the pooled sample; (**B**) coproculture after treatment: *H. contortus* third-stage larvae (L3) in the pooled sample (400x magnification; Lugol’s iodine staining in both images).

The results of the individual faecal egg count (FEC) are presented in detail in Table 1. The FECR_1_ was **−88%** (CI 95%: −248%, −1%) and the FECR_2_ was **−202%**, both indicating the resistance of GINs to EPM. The examination of the coproculture prepared from the pooled sample before treatment revealed the presence of *T. circumcincta*, *H. contortus*, and *Oesophagostomum spp.*, whereas in the following coproculture treatment, only *H.contortus* was found (Figure 1).

### 3.2. EHT

Hatching of eggs was observed in all the wells regardless of TBZ concentration. Percentage of eggs hatching at the DC (0.1 μg/mL) was **96.2%** (CI 95%: 92.6%, 98.1%). The IC_50_ value was **221.1** μg/mL (CI 95%: 214.2, 227.9 μg/mL), which is significantly above the DC threshold value which indicates resistance to BZs (Table 2).

### 3.3. LDT

Larval development was observed in all wells regardless of the anthelmintics’ concentrations in the case of TBZ and IVM-AG, whereas LEV proved to be effective and stopped larval development at the threshold concentration. It was considered that the herd harboured resistant GINs if L3 larvae development was observed at DC threshold (TBZ—0.08 μg/mL; IVM-AG—21.6 ng/mL; LEV—2 μg/mL). Infectious L3 development at DC were of **94.9%** (CI 95%: 90.0%, 97.4%) for TBZ, **94.8%** (CI 95%: 90.5%, 97.2%) for IVM-AG, and **0%** (CI 95%: 0%, 10.4%) for LEV. These results indicate high resistance to TBZ and IVM-AG. Detailed results of LDT are presented in Table 3. *H. contortus* L3 larvae was the only species found in the wells with the highest concentrations of TBZ and IVM-AG, respectively.

## 4. Discussion

Our study is the first report of AR in GINs in the goat population in Romania. Resistance was detected to two classes of anthelmintics: MLs and BZs. We employed two types of diagnostic methods to confirm AR [46]. The FECRT, an in vivo AR diagnostic method, was used to evaluate the efficacy of EPM. The results of this test were analysed using two different calculative methods that are applied only in treated animals (without the control group) [50]. One method evaluated the FECR of the treated herd (group) based on average values, and the other evaluated the FECR using individual evaluations in treated goats (before and after treatment). Furthermore, two in vitro methods were used: EHT for the detection of resistance to BZs, and LDT for detection of resistance to all three classes of anthelmintics. The interpretation of the results of these in vitro methods depends on the DC threshold values selected [7]. We decided to use the DC rather than the LC_50_ criterion for differentiation between resistant and susceptible GINs. Results of previous studies suggested that application of the LC_50_ may sometimes underestimate the resistance and possibly other herds might have been incorrectly classified by the criterion as susceptible [51]. It has been suggested that using the IC_99_/LC_99_ or the DC values in the in vitro tests such as EHT or LDT can substantially increase the sensitivity and identify resistance when only a small proportion of the GINs population is resistant [35,37,51,52,53]. A similar resistance classification criterion was successfully implemented in AR field studies [7,14,21,22,24,25,48]. For the present study, we adopted threshold values accepted in the scientific literature.

The results of FECRT carried out in this study indicate the resistance of GINs to EPM with both calculative methods (FECR_1_ = −88% and FECR_2_ = −202%). In addition, the results obtained for IVM-AG in LDT also indicate resistance to drugs from MLs group, especially avermectins. Similarly, the results of in vitro methods (EHT and LDT) indicate resistance to BZs in this herd. In both tests, IC_50_ and LC_50_ values were much higher than the DC threshold values and L3 larvae development or hatching of eggs in the threshold concentrations were also on a very high level. LEV was the only drug that stopped the development of L3 larvae 100% (CI 95%: 89.6%, 100%) at the DC threshold in LDT. Based on the in vivo and in vitro tests results and deworming history of the herd, we assume that only LEV remained the only effective anthelmintic against GINs in the examined herd, *H. contortus* was the only nematode species found in coproculture after EPM treatment. Furthermore, *H. contotus* L3 larvae was the only species found in the wells with the highest concentrations of TBZ and IVM-AG, respectively. This suggests that resistance to both BZs and MLs was present for the aforementioned nematode species.

The BZs have been most widely used worldwide since the successful production of thiabendazole in the 1960s. Nowadays, the resistance of BZs has been reported worldwide [54]. In our study, we chose the EHT and LDT for the detection of BZs resistance because these methods have been suggested to have a higher sensitivity than FECRT in detecting BZ resistance [35]. FECRT is considered reliable only if more than 25% of the population of nematodes are resistant [55]. There are several studies indicating that FECRT could underestimate low levels of BZ resistance. Crook et al. [36] showed the disagreement between the results of FECRT and LDT on two goat farms, where susceptibility to albendazole were indicated by FECRT and LDT indicated resistance to BZs. In the survey of Diez-Banos et al. [56] performed on sheep flocks, the BZs resistance using FECRT was found in 18% (13) of flocks, whereas 29% (21) of flocks were found to be resistant to BZs when EHT was used. A similar study performed by Babják et al. [21] pointed to a moderate correlation between in vivo and in vitro tests for detecting BZs resistance among the 30 goat farms. Moreover, the EHT and LDT have the greater potential to detect low levels of AR by using DC to reduce the number of drug concentrations required and to increase the sensitivity of the tests [5]. It allows the reliable detection of a frequency of resistance alleles below 10% [57] and is fairly reliable for the detection of BZ resistance under field conditions [35,37,53].

Thus far, the resistance to MLs has been reported in Europe in goat herds from Denmark [58], Czech Republic [22], Scotland [59], Slovakia [34,60], Switzerland [17,61,62,63], Germany [63], and Poland [7,24,25] on a timespan of almost 30 years. This implies that the ML resistance might be spread throughout Europe, but its prevalence is still at a lower level than in case of BZs [2,6].

In Romania, albendazole/fenbendazole (BZs) and ivermectin/eprinomectin (MLs) are the most frequently used anthelmintics in small ruminants, while levamisole is used only sporadically (inference from personal experience). BZs are relatively cheap and the withdrawal period is short, which is of great importance for the farmers as they are mainly interested in obtaining dairy products from their herds. As for MLs, ivermectin is the most commonly used anthelmintic in small ruminants, despite the extended withdrawal time for milk. EPM has more recently been approved for use in goats and rapidly gained in popularity thanks to its easy administration (pour-on), being the only anthelmintic with no milk-withdrawal period authorised in goats. However, for many Romanian farmers, the financial aspect is the one that prevails. Thus far, no data are available in Romania regarding the resistance to any anthelmintics in goats or sheep. The only study about AR in Romania was performed on horses in 2015 [38].

The resistance to EPM found in this herd was unexpected since the drug has never been used in the herd before. However, this could be explained by the fact that eprinomectin and ivermectin, as avermectins (MLs), share the same mechanism of action [64], the latter being one of the most widely used drugs for controlling GINs in goats from Romania. Another explanation of the resistance to EPM in this herd could be attributed to the cross-resistance between BZs and ML. The same nucleotide changes in IVM resistant *H. contortus* have been observed in the gene coding for B-tubulin isotype 1, which is responsible for resistance in BZs, even though these chemical groups have different mechanisms of action [65]. Both explanations seem possible since the goats from the studied herd were only treated with ivermectin and albendazole in the last few years. Levamisole is very rarely used in goats in Romania. This most likely coincides with the fact that, to date, no commercial product based on levamisole has been approved for usage in goats. The herd from this study had never been treated with LEV before and the results of LDT showed susceptibility of GINs to this drug.

There are several factors limiting the effective use of anthelmintics. One of the most important factors contributing to the rapid development of AR is underdosing [66]. In Romania, the anthelmintics commonly applied by farmers are not licensed for goats so they frequently extrapolate the dosage from other species. The goats metabolise anthelmintic medications more rapidly than other species, so they require higher doses of drugs [67]. However, our experience shows that the farmers either do not have knowledge about these requirements or choose to neglect them due to financial reasons. The situation was similar in the herd under study, where the owner dosed the drug as indicated for sheep. Cringoli et al. [68] observed big differences in FECR% between a 0.5 mg/kg/bw single dose (dosage recommended for sheep) and 1 mg/kg/bw double dose of EPM. Other factors that may contribute to the AR are the following: constant and repeated use of an anthelmintic belonging to one chemical group, too frequent deworming, and a lack of quarantine of newly purchased animals [69].

From our field experience, we know that very few goat farmers ask for faecal examination needed for the diagnosis of GINs. The veterinary practitioners and farmers should be educated about all the aspects of AR and advised on how they can implement efficient control strategies against GINs in goats.

## 5. Conclusions

This study provides the first report of AR to two classes of anthelmintics: MLs and BZs, in goats in Romania. Considering the increasing prevalence of AR in goat herds in Europe and around the globe, we believe that the findings of our study on AR in goats in Romania do not represent a singular event and could hence be just the evidence of a much larger problem.

## Figures and Tables

**Table 1:** Individual faecal egg count (FEC) given as eggs per gram before and after treatment.

Goat No. (>6 Months)	Pre-Treatment (Day 0)	Post-Treatment (Day 14)
1	1600	2100
2	550	6950
3	150	1000
4	2350	2950
5	1600	1400
6	750	1350
7	1350	2850
8	1750	2500
9	700	1050
10	1500	950
Mean ± SD ^a^	1230 ± 669	2310 ± 1802
Median	1425	1750
IQR ^b^ (range)	700–1600 (150–2350)	1050 −2850 (950–6950)
FECR_1_ ^c^ (CI 95%)	−88% (CI 95%: −248, −1)	
FECR_2_ ^c^	–202%	

^a^ Standard deviation; ^b^ interquartile range; ^c^ faecal egg count reduction.

**Table 2:** Results of egg hatch test (EHT).

	TBZ
**IC_50_**	221.1 μg/mL
**IC_99_**	undeterminable *
**Percentage of eggs hatching at the DC (0.1 μg/mL) (CI 95%)**	96.2% (CI 95%: 92.6, 98.1)

IC = inhibition concentration; DC = discriminating concentration; * infinitely high; TBZ = thiabendazole.

**Table 3:** Results of larval development test (LDT).

	TBZ	IVM-AG	LEV
**LC_50_**	Undeterminable *	121.5 ng/mL	0.082 µg/mL
**LC_90_**	Undeterminable *	315.9 ng/mL	0.154 µg/mL
**LC_99_**	Undeterminable *	895.9 ng/mL	0.305 µg/mL
**Corrected % inhibited at DC(CI 95%)**	5.1% (2.6%, 10.0%)	5.2% (2.8%, 9.5%)	100% (89.6%, 100%)

LC = lethal concentration; DC = discriminating concentration; * infinitely high; TBZ = thiabendazole; IVM-AG = ivermectin aglycone; LEV = levamisole.

## Data Availability

The data presented in this study are available in the scientific publications listed in the Reference section.
